# Effects of lifestyle-related factors on ischemic heart disease according to body mass index and fasting blood glucose levels in Korean adults

**DOI:** 10.1371/journal.pone.0216534

**Published:** 2019-05-15

**Authors:** Jiae Shin, Dongwoo Ham, Sangah Shin, Seul Ki Choi, Hee-Young Paik, Hyojee Joung

**Affiliations:** 1 Department of Public Health Science, Graduate School of Public Health, Seoul National University, Seoul, Republic of Korea; 2 Institute of Health and Environment, Seoul National University, Seoul, Republic of Korea; 3 Department of Food and Nutrition, School of Food Science and Technology, Chung-Ang University, Anseong, Gyeonggi-do, Republic of Korea; 4 Department of Health Promotion, Education, and Behavior, Arnold School of Public Health, University of South Carolina, Columbia, South Carolina, United States of America; 5 Centered for Gendered Innovation in Science and Technology Research (GISTeR), Korea Federation of Women’s Science & Technology Associations, Seoul, Republic of Korea; Medical University of Vienna, AUSTRIA

## Abstract

**Objective:**

To investigate the effects of lifestyle-related factors on ischemic heart disease (IHD) according to body mass index (BMI) and fasting blood glucose (FBG) levels among Korean adults.

**Methods:**

A total of 119,929 men and 89,669 women (from National Sample Cohort version 2.0, National Health Insurance Service) aged ≥20 years who were examined during 2003–2006 and had no preexisting type 2 diabetes or circulatory diseases were followed until December 2015 to confirm IHD incident cases. Data on lifestyle-related factors (BMI, FBG, diet, smoking, alcohol consumption, and physical activity) were collected at baseline. Lifestyle-related risk factors were defined as mainly vegetable/mainly meat diet, former/current smoking, alcohol consumption ≥3 times/week, and no physical activity. Associations between these factors and IHD were examined using Cox proportional hazards regression models.

**Results:**

High BMI (≥25 kg/m^2^), high FBG (≥100 mg/dL), mainly meat diet, and former/current smoking were associated with increased risk for IHD. Alcohol consumption ≤twice/week and physical activity ≤twice/week were associated with lower risk of IHD. With increased lifestyle-related risk factors, the risk of IHD also increased in women (hazard ratio [HR] = 3.21, 95% confidence interval [CI] 2.18–4.73) and men (HR = 1.66, 95% CI 1.48–1.85). This increase was larger in women, with a significant sex interaction (p = 0.0001). Significant interactions between BMI and alcohol consumption (p = 0.0002) and between BMI and physical activity (p = 0.0063) were observed. Interactions were seen between FBG level and meal type in both BMI<25 kg/m^2^ (p = 0.0106) and BMI≥25 kg/m^2^ (p = 0.0281) and between FBG level and alcohol consumption in BMI ≥25 kg/m^2^ (p = 0.0118).

**Conclusions:**

The impact of lifestyle-related factors on IHD was more pronounced in women than in men and may be modified by BMI and FBG level among Korean adults. This might be taken into account when planning individual interventions to reduce IHD risk.

## Introduction

Ischemic heart disease (IHD), also known as coronary heart disease, is one of the leading causes of death globally [1, 2]. In South Korea (hereafter “Korea”), IHD contributes to >50% of deaths (31.0 cases in men and 26.3 cases in women per 100,000 persons) from cardiovascular diseases (CVDs), which were the second biggest causes of death (58.2 cases per 100,000 persons) after cancer in 2016 [3, 4]. Although more common among men [2, 5], IHD is one of the major causes of death in both men and women [6]. Women seem to have IHD events 7–10 years later than men, yet the outcome of IHD is worse in women [7, 8].

The morbidity and mortality rates associated with IHD among people with obesity or impaired glucose tolerance were reported to be higher than those of healthy people [9–13]. Furthermore, high body mass index (BMI) and fasting blood glucose (FBG) levels are affected by other lifestyle-related factors such as diet, smoking, alcohol consumption, and physical activity [14–22]. People who are overweight or obese were found to have a higher IHD risk than those with normal BMI. Additionally, risk factors related to unhealthy lifestyle increased IHD risk. For example, heavy smoking and sedentary lifestyle were strongly related to IHD risk in people with an increased BMI [9]. Meanwhile, a study conducted in Japan reported that high blood glucose levels increased CVD mortality more than obesity in combination with 2 or more metabolic factors, such as BMI>25 kg/m^2^, high blood pressure, high blood glucose, high triglyceride, and low high-density lipoprotein cholesterol (HDL-C) levels [12]. Moreover, FBG levels strongly interacted with the other lifestyle-related factors in intervention studies on IHD risk; a lower calorie intake and more vigorous physical activity decreased FBG levels [23, 24]. To our knowledge, however, there is limited evidence about the effects of the interaction between BMI or FBG levels and the other lifestyle-related factors on the incidence of IHD among Koreans. Furthermore, the association between lifestyle-related factors and IHD has not been investigated in large-scale cohorts in Korea.

Most studies on IHD considering the association between BMI or FBG levels and the other lifestyle-related factors have been conducted in Western countries [9, 10, 15, 23–25]. Hence, independent studies on IHD risk for Asian countries would be necessary considering that Asians have different ethnicity and standards for obesity as well as different lifestyle-related factors, which may affect IHD risk [11, 26–28]. Therefore, this study aimed to assess the effects of lifestyle-related factors on IHD incidence according to BMI and FBG levels among Korean adults.

## Materials and methods

This study was approved by the Institutional Review Board of Seoul National University (IRB No. E1709/002-001) and the National Health Insurance Service (NHIS) (No. NHIS-2017-2-541).

### Data source

The National Sample Cohort version 2.0 (NSC-v2) of the NHIS was used for this analysis. Korea established the NHIS in 1963, and all citizens have been registered under a universal health insurance system since 1989 [29]. All national health insurance records from healthcare institutions are gathered by the NHIS. In 2017, NHIS constructed NSC-v2 by extracting 1 million representative samples from the total eligible Korean population in 2006. The need for prior consent from the study population was waived because data were anonymized by NHIS before entry into the database, according to their strict rules to protect personal information. The cohort has five databases: birth and death, insurance eligibility, medical treatment, general health examination, and information on healthcare providers between 2002 and 2015. More details have been described elsewhere [29–31].

### Study subjects

Among the 1 million samples from NSC-v2, 313,156 people who received a general health examination at least once between 2003 and 2006 were initially selected for analysis. We excluded subjects who had medical records of type 2 diabetes (T2D) or circulatory diseases before the health examinations (n = 90,375) based on International Classification of Diseases (ICD)-10 codes [32]: E11 for T2D and I00-I99 for circulatory diseases. We also excluded subjects aged <20 years (n = 1,013); those who could be considered as outliers (with a mean of ±3 standard deviations) [33] or had missing information on lifestyle-related factors including BMI, FBG, meal type, smoking status, alcohol consumption, and physical activity (n = 12,169); or those with no information on insurance eligibility (n = 1) at baseline. A total of 209,598 people (119,929 men and 89,669 women) were included in the final analysis.

### Variables

We linked the five databases of NSC-v2 using anonymized identity numbers. Age at baseline was calculated by subtracting the year of birth from the year of health examination and was categorized into three groups: 20–39, 40–59, and ≥60 years. Income level was estimated according to the percentiles of health insurance fee and was categorized into three groups: high (upper 30%), medium (31–70%), and low (lower 30% or medical-aid beneficiaries).

Height, weight, and biochemical indices after an overnight fasting were obtained from the health examination records. The subjects were stratified according to BMI and FBG levels. BMI was calculated from the measurement of height and weight and categorized into two groups: <25.0 and ≥25.0 kg/m^2^ [11, 26]. FBG was stratified into two levels: normal (<100 mg/dL) and high (≥100 mg/dL) [34]. Risk status was divided into four groups according to BMI (kg/m^2^) and FBG (mg/dL) levels: (1) BMI<25 and FBG<100, (2) BMI<25 and FBG≥100, (3) BMI≥25 and FBG<100, and (4) BMI≥25 and FBG≥100.

Data on lifestyle-related factors were collected from self-reported questionnaires during the health examinations. The question on meal types consisted of three options: balanced consumption of vegetables and meat, consuming mainly vegetables, and consuming mainly meat. Smoking was classified based on smoking status: never, former, or current smokers. Alcohol consumption frequency was categorized into three levels: none, ≤twice/week, or ≥3 times/week [35]. Physical activity frequency was assessed on weekly basis and classified into three groups: none, ≤twice/week, or ≥3 times/week [36]. Lifestyle-related risk factors were defined as follows: mainly vegetable/mainly meat diet, former/current smoking, alcohol consumption ≥3 times/week, or no physical activity. IHD incidence was identified based on ICD-10 codes (I20-I25) and dates of the first diagnosis in the medical treatment database [32].

### Statistical analysis

The subjects’ medical records were followed from the first health examination until the first diagnosis of IHD, loss of insurance eligibility, or end of follow-up (December 2015). Person-time for each subject was calculated as the number of months of follow-up divided by 12 to convert the value into a fraction of years. We calculated frequencies and percentages for each of the following variables: sex, age, income level, BMI, FBG, meal type, smoking status, alcohol consumption, and physical activity at baseline. Chi-square tests were performed to determine whether the distributions of lifestyle-related factors between men and women were significantly different. The association between lifestyle-related factors (BMI, FBG, meal type, smoking, alcohol consumption, and physical activity) and IHD incidence was estimated in hazard ratios (HR) from Cox proportional hazards regression models according to sex and risk status (BMI and FBG levels). Population attributable risk (PAR) was calculated using the equation *PAR* = *P*(*HR*−1)÷[1+*P*(*HR*−1)], where P is the prevalence of exposure [37]. The assumption of proportional hazards was evaluated and satisfied by examining Schoenfeld residuals [38]. Confounding variables including age, sex (for total subjects only), BMI, FBG, income level, meal type, smoking, alcohol consumption, and physical activity were adjusted. We computed P_interaction_ values using a likelihood ratio test to compare Cox proportional hazard models with and without cross-product terms for IHD incidence, and each lifestyle category in the analyses was stratified by sex, or by BMI and FBG [39]. All statistical analyses were performed with SAS Enterprise Guide version 7.1 (SAS Institute, Cary, NC). P-values of <0.05 were considered statistically significant.

## Results

The IHD incidence rate in men (1359.9 cases per 100,000 person-years) was significantly higher than that in women (1336.0 cases per 100,000 person-years; HR = 0.91, 95% confidence interval [CI] 0.88–0.94) during the follow-up periods. The average follow-up periods were 10.5 and 10.3 person-years in men and women, respectively.

### Baseline characteristics of study subjects

Table 1 shows the general characteristics of the subjects at baseline. The distributions of the subjects were significantly different for all variables between men and women (p<0.0001). More men had a high BMI (≥25 kg/m^2^; 32.5%) and a high FBG level (≥100 mg/dL; 24.7%) than women (19.4% and 16.4%, respectively). Regarding risk status, more men (9.6%) had a risk status of a high BMI and a high FBG than women (4.7%). Balanced diet was the most common in both men (77.9%) and women (71.9%). The proportions of never smokers and non-drinkers were greater in women than in men. More women (65.1%) had sedentary lifestyles than men (47.9%).

**Table 1:** General characteristics of the subjects at the baseline.

Variables	Total		Men		Women		P value^a^
	n	%	n	%	n	%	
**N**	209,598		119,929		89,669		
**Age (years)**							
20–39	97,128	46.3	61,950	51.7	35,178	39.2	<0.0001
40–59	91,334	43.6	47,168	39.3	44,166	49.3	
≥60	21,136	10.1	10,811	9.0	10,325	11.5	
**Income level**^**b**^							
Low	62,978	30.0	29,271	24.4	33,707	37.6	<0.0001
Medium	83,786	40.0	52,091	43.4	31,695	35.3	
High	62,834	30.0	38,567	32.2	24,267	27.1	
**BMI (kg/m**^**2**^**)**							
<25	153,305	73.1	80,997	67.5	72,308	80.6	<0.0001
≥25	56,293	26.9	38,932	32.5	17,361	19.4	
**FBG (mg/dL)**							
<100	165,265	78.8	90,330	75.3	74,935	83.6	<0.0001
≥100	44,333	21.2	29,599	24.7	14,734	16.4	
**Risk status**							
BMI<25, FBG <100	124,628	59.5	62,869	52.4	61,759	68.9	<0.0001
BMI<25, FBG≥100	40,637	19.4	27,461	22.9	13,176	14.7	
BMI≥25, FBG <100	28,677	13.7	18,128	15.1	10,549	11.8	
BMI≥25, FBG≥100	15,656	7.5	11,471	9.6	4,185	4.7	
**Meal type**							
Balanced	157,826	75.3	93,389	77.9	64,437	71.9	<0.0001
Mainly vegetables	38,626	18.4	17,404	14.5	21,222	23.7	
Mainly meat	13,146	6.3	9,136	7.6	4,010	4.5	
**Smoking**							
Never	128,409	61.3	43,168	36.0	85,241	95.1	<0.0001
Former	17,658	8.4	16,151	13.5	1,507	1.7	
Current	63,531	30.3	60,610	50.5	2,921	3.3	
**Alcohol consumption**							
None	97,879	46.7	35,700	29.8	62,179	69.3	<0.0001
≤twice/week	91,880	43.8	66,263	55.3	25,617	28.6	
≥3 times/week	19,839	9.5	17,966	15	1,873	2.1	
**Physical activity**							
None	115,868	55.3	57,502	47.9	58,366	65.1	<0.0001
≤twice/week	58,375	27.9	40,690	33.9	17,685	19.7	
≥3 times/week	35,355	16.9	21,737	18.1	13,618	15.2	

^a^P values were obtained from Chi-square test.

^b^Low: lower 30 percentiles or medical-aid beneficiaries; medium: 31–70 percentiles; high: upper 30 percentiles

Abbreviations: BMI, body mass index; FBG, fasting blood glucose

### HRs and PARs of IHD according to lifestyle-related factors

The HRs and PARs of IHD incidence among men and women according to lifestyle-related factors are presented in Table 2. Among lifestyle-related factors of IHD, high BMI (≥25 kg/m^2^) exhibited the highest PAR both in men (8.39%) and women (6.82%). In all subjects, those with a high BMI or a high FBG level (≥100 mg/dL) showed a significantly increased IHD risk compared to those with normal BMI or FBG levels (HR = 1.31, 95% CI 1.28–1.34, and HR = 1.10, 95% CI 1.07–1.13, respectively). Subjects with a mainly meat diet (HR = 1.15, 95% CI 1.09–1.21), former (HR = 1.08, 95% CI 1.04–1.13), and current smokers (HR = 1.09, 95% CI 1.06–1.13) had a significantly increased IHD incidence, whereas subjects who consumed alcohol ≤twice/week (HR = 0.89, 95% CI 0.87–0.92) and performed physical activity ≤twice/week (HR = 0.97, 95% CI 0.94–0.99) showed a decreased IHD risk. Among men, those with mainly meat diet showed an increased IHD risk and those with physical activity ≤twice/week had a reduced risk (HR = 1.17, 95% CI 1.10–1.24, and HR = 0.96, 95% CI 0.93–0.99, respectively).

**Table 2:** Hazard ratios and population attributable risk of ischemic heart disease according to lifestyle-related factors.

	Total					Men					Women					P_interaction_^b^
	Cases	Person-year	HR^a^	95% CI	PAR %	Cases	Person-year	HR^a^	95% CI	PAR %	Cases	Person-year	HR^a^	95% CI	PAR %	
**BMI (kg/m**^**2**^**)**																
<25	19,430	1,610,884	1.00	Ref.		10,705	856,239	1.00	Ref.		8,725	754,645	1.00	Ref.		0.1949
≥25	10,033	577,557	1.31	(1.28, 1.34)	8.06	6,374	405,232	1.29	(1.25, 1.33)	8.39	3,659	172,326	1.30	(1.25, 1.35)	6.82	
**FBG (mg/dL)**																
<100	21,664	1,736,649	1.00	Ref.		11,949	957,509	1.00	Ref.		9,715	779,141	1.00	Ref.		0.2753
≥100	7,799	451,792	1.10	(1.07, 1.13)	2.41	5,130	303,962	1.10	(1.07, 1.14)	2.73	2,669	147,830	1.10	(1.05, 1.14)	1.96	
**Meal type**																
Balanced	21,869	1,649,931	1.00	Ref.		13,231	982,272	1.00	Ref.		8,638	666,659	1.00	Ref.		
Mainly vegetables	6,018	400,816	1.01	(0.98, 1.04)	0.20	2,640	182,935	0.98	(0.94, 1.03)	-0.32	3,378	217,881	1.03	(0.99, 1.07)	0.79	0.0012
Mainly meat	1,576	138,694	1.15	(1.09, 1.21)	0.70	1,208	96,264	1.17	(1.10, 1.24)	1.03	368	42,430	1.09	(0.98, 1.21)	0.25	
**Smoking**																
Never	18,225	1,332,368	1.00	Ref.		6,457	450,451	1.00	Ref.		11,768	881,917	1.00	Ref.		
Former	2,748	184,881	1.08	(1.04, 1.13)	0.69	2,572	169,257	1.07	(1.02, 1.12)	0.99	176	15,624	1.19	(1.02, 1.38)	0.23	0.0174
Current	8,490	671,192	1.09	(1.06, 1.13)	2.38	8,050	641,763	1.06	(1.03, 1.10)	2.67	440	29,429	1.20	(1.09, 1.33)	0.59	
**Alcohol consumption**																
None	15,497	1,005,517	1.00	Ref.		5,781	369,071	1.00	Ref.		9,716	636,446	1.00	Ref.		
≤twice/week	10,426	981,063	0.89	(0.87, 0.92)	-4.37	8,041	709,587	0.89	(0.86, 0.92)	-5.82	2,385	271,488	0.91	(0.87, 0.96)	-1.90	0.0902
≥3 times/week	3,540	201,850	1.00	(0.96, 1.04)	-0.04	3,257	182,813	1.01	(0.96, 1.05)	0.19	283	19,037	1.05	(0.93, 1.19)	0.11	
**Physical activity**																
None	16,546	1,203,047	1.00	Ref.		8,525	598,827	1.00	Ref.		8,021	604,220	1.00	Ref.		
≤twice/week	7,432	618,269	0.97	(0.94, 0.99)	-0.78	5,196	434,438	0.96	(0.93, 0.99)	-1.27	2,236	183,831	0.97	(0.92, 1.01)	-0.56	0.6468
≥3 times/week	5,485	367,125	0.99	(0.96, 1.02)	-0.19	3,358	228,206	0.98	(0.94, 1.02)	-0.40	2,127	138,919	0.98	(0.94, 1.03)	-0.35	
**Total numbers of risk factors**^**c**^																
No risk factors	2,560	236,044	1.00	Ref.		978	85,877	1.00	Ref.		1,582	150,167	1.00	Ref.		
1–2 risk factors	18,474	1,452,546	1.14	(1.09, 1.19)	7.60	9,534	767,606	1.10	(1.03, 1.17)	4.94	8,940	684,941	1.15	(1.09, 1.21)	9.36	0.0001
3–4 risk factors	7,927	476,557	1.35	(1.29, 1.41)	14.50	6,091	385,315	1.29	(1.21, 1.38)	12.96	1,836	91,242	1.37	(1.28, 1.47)	13.38	
5–6 risk factors	502	23,294	1.74	(1.58, 1.92)	15.32	476	22,673	1.66	(1.48, 1.85)	14.06	26	620	3.21	(2.18, 4.73)	13.52	

^a^HRs were adjusted for age, sex (for total subjects only), income level, and additionally mutually adjusted for lifestyle-related factors (all categorical variables).

^b^P_interaction_: p value of interaction between lifestyle-related factor and sex

^c^Risk factors were defined as BMI≥25 kg/m^2^, FBG≥100 mg/dL, mainly vegetable/mainly meat meal diet, former/current smoking, ≥3 times/week alcohol consumption, or no physical activity; and HRs were adjusted for age, sex (for total subjects only), and income level (all categorical variables).

Abbreviations: HR, hazard ratio; CI, confidence interval; PAR, population attributable risk; BMI, body mass index; FBG, fasting blood glucose

In addition, the associations between lifestyle-related factors and IHD incidence were modified by sex, with significant interactions with meal types (p = 0.0012) and smoking status (p = 0.0174). A significantly increased risk of IHD was identified in both men and women, as the numbers of lifestyle-related risk factors increased. Comparing subjects with 5–6 risk factors with subjects without any lifestyle-related risk factors, the HR for IHD incidence was 1.74 (95% CI 1.58–1.92) in total subjects. Noticeably, this association was stronger in women (HR = 3.21, 95% CI 2.18–4.73) than in men (HR = 1.66, 95% CI 1.48–1.85), with a significant sex interaction (p = 0.0001 for interaction).

**Fig 1:**
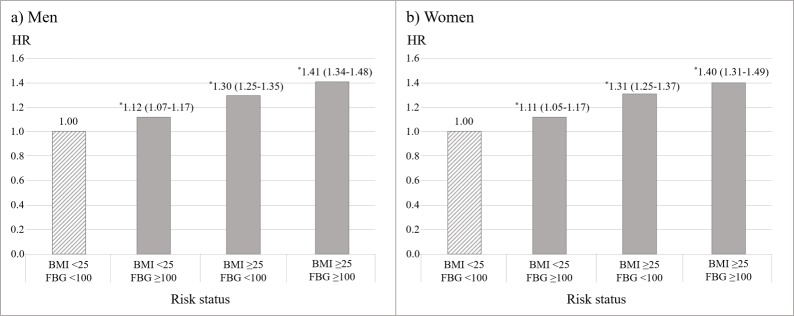
Hazard ratios of ischemic heart disease according to BMI and FBG levels. *95% confidence interval of hazard ratio does not include the reference value (1.00). HRs of IHD were adjusted for age, income level, meal type, smoking status, alcohol consumption, and physical activity (all categorical variables). A group of BMI<25 kg/m^2^ and FBG<100 mg/dL was set as reference and colored as a pattern in the bar graph. Abbreviations: HR, hazard ratio; BMI, body mass index (kg/m^2^); FBG, fasting blood glucose (mg/dL).

Men and women showed similar patterns of HRs of IHD incidence according to risk status of BMI and FBG levels (Fig 1). Compared to those with normal BMI and FBG levels, both men and women with high BMI and FBG levels showed the highest HRs of 1.41 (95% CI 1.34–1.48) and 1.40 (95% CI 1.31–1.49), respectively.

### HRs of IHD according to lifestyle-related factors by the status of BMI and FBG levels

HRs of IHD incidence according to individual lifestyle-related factors by the status of BMI and FBG levels are presented in Table 3. A mainly vegetable meal type was positively associated with IHD risk in subjects with BMI <25 kg/m^2^ and FBG <100 mg/dL (HR = 1.05, 95% CI 1.01–1.09). A meal type consisting of mainly meat was positively associated with IHD risk, except in subjects with BMI ≥25 kg/m^2^ and FBG <100 mg/dL. Former (HR = 1.09, 95% CI 1.03–1.16) and current (HR = 1.08, 95% CI 1.04–1.13) smoking significantly increased the IHD risk among subjects with BMI <25 kg/m^2^, which remained significant in subgroup analysis stratified by FBG. Alcohol consumption ≤twice/week reduced the incidence of IHD, but not significantly among subjects with BMI ≥25 kg/m^2^ and FBG ≥100 mg/dL. Alcohol consumption ≥3 times/week significantly increased IHD risk (HR = 1.22, 95% CI 1.09–1.36), while physical activity ≥3 times/week decreased the risk (HR = 0.90, 95% CI 0.82–0.99) among subjects with BMI ≥25 kg/m^2^ and FBG ≥100 mg/dL. Compared to the results of total subjects, men showed similar patterns of the association between lifestyle-related factors and IHD risk regardless BMI and FBG levels, but with a varied significance. Conversely, only women with normal BMI and FBG levels had consistent associations between IHD risk and current smoking and alcohol consumption ≤twice/week with the results of total subjects.

**Table 3:** Hazard ratios of ischemic heart disease according to lifestyle-related factors stratified by the status of BMI and FBG levels.

	BMI < 25													BMI ≥ 25													P _interaction_^d^
	Cases	Person-year	HR^a^	95% CI	FBG < 100				FBG ≥ 100				P _interaction_^c^	Cases	Person-year	HR^a^	95% CI	FBG < 100				FBG ≥ 100				P _interaction_^c^	
					Cases	Person-year	HR^b^	95% CI	Cases	Person-year	HR^b^	95% CI						Cases	Person-year	HR^b^	95% CI	Cases	Person-year	HR^b^	95% CI		
** Total**																											
**Meal type**																											
Balanced	14,084	1,197,496	1.00	Ref.	10,614	977,369	1.00	Ref.	3,470	220,127	1.00	Ref.		7,785	451,436	1.00	Ref.	5,356	327,728	1.00	Ref.	2,429	123,708	1.00	Ref.		
Mainly vegetables	4,430	320,896	1.02	(0.99, 1.06)	3,468	262,087	1.05	(1.01, 1.09)	962	58,809	0.94	(0.88, 1.01)		1,588	79,909	0.99	(0.94, 1.04)	1,101	58,905	0.97	(0.91, 1.04)	487	21,004	1.03	(0.94, 1.14)		
Mainly meat	1,916	92,481	1.20	(1.12, 1.28)	1,707	77,189	1.20	(1.12, 1.30)	209	15,292	1.17	(1.01, 1.34)	0.0106	660	46,212	1.08	(0.999, 1.174)	418	33,372	1.03	(0.93, 1.14)	242	12,840	1.19	(1.04, 1.36)	0.0281	0.0614
**Smoking**																											
Never	12,310	1,021,279	1.00	Ref.	9,667	854,329	1.00	Ref.	2,643	166,950	1.00	Ref.		5,915	311,090	1.00	Ref.	4,138	230,104	1.00	Ref.	1,777	80,986	1.00	Ref.		
Former	1,651	121,791	1.09	(1.03, 1.16)	1,171	94,743	1.08	(1.01, 1.15)	480	27,048	1.14	(1.02, 1.27)		1,097	63,089	1.07	(0.99, 1.15)	735	43,981	1.11	(1.02, 1.21)	362	19,108	0.99	(0.88, 1.12)		
Current	5,469	467,814	1.08	(1.04, 1.13)	3,951	367,573	1.07	(1.02, 1.12)	1,518	100,241	1.11	(1.02, 1.20)	0.6033	3,021	203,378	1.09	(1.03, 1.15)	2,002	145,921	1.10	(1.03, 1.18)	1,019	57,457	1.07	(0.97, 1.17)	0.1261	0.0925
**Alcohol consumption**																												
None	10,637	764,894	1.00	Ref.	8,331	637,063	1.00	Ref.	2,306	127,831	1.00	Ref.		4,860	240,622	1.00	Ref.	3,486	179,788	1.00	Ref.	1,374	60,834	1.00	Ref.		
≤twice/week	6,569	709,321	0.87	(0.84, 0.90)	5,034	581,869	0.88	(0.85, 0.92)	1,535	127,452	0.85	(0.79, 0.92)		3,857	271,742	0.95	(0.90, 0.99)	2,637	197,862	0.94	(0.884, 0.995)	1,220	73,880	0.97	(0.88, 1.06)		
≥3 times/week	2,224	136,657	0.96	(0.91, 1.01)	1,424	97,713	0.94	(0.887, 1.003)	800	38,944	1.00	(0.91, 1.09)	0.2102	1,316	65,194	1.08	(1.01, 1.15)	752	42,356	1.00	(0.92, 1.09)	564	22,838	1.22	(1.09, 1.36)	0.0118	0.0002
**Physical activity**																											
None	11,263	916,522	1.00	Ref.	8,554	752,500	1.00	Ref.	2,709	164,022	1.00	Ref.		5,283	286,513	1.00	Ref.	3,589	208,329	1.00	Ref.	1,694	78,184	1.00	Ref.		
≤twice/week	4,649	437,696	1.01	(0.97, 1.05)	3,529	355,897	0.97	(0.93, 1.01)	1,120	81,799	0.96	(0.89, 1.03)		2,783	180,572	0.97	(0.92, 1.01)	1,911	131,255	0.98	(0.93, 1.04)	872	49,317	0.94	(0.86, 1.02)		
≥3 times/week	3,518	256,653	0.88	(0.85, 0.92)	2,706	208,247	1.02	(0.98, 1.07)	812	48,406	0.98	(0.90, 1.06)	0.2655	1,967	110,473	0.95	(0.899, 0.999)	1,375	80,421	0.97	(0.91, 1.03)	592	30,052	0.90	(0.82, 0.99)	0.1213	0.0063
**Men**																											
**Meal type**																											
Balanced	8,150	659,284	1.00	Ref.	5,825	514,819	1.00	Ref.	2,325	144,465	1.00	Ref.		5,081	322,989	1.00	Ref.	3,363	229,616	1.00	Ref.	1,718	93,373	1.00	Ref.		
Mainly vegetables	1,909	140,563	0.98	(0.94, 1.04)	1,431	109,137	1.03	(0.97, 1.09)	478	31,426	0.88	(0.80, 0.97)		731	42,361	0.98	(0.91, 1.07)	486	30,243	0.97	(0.88, 1.07)	245	12,118	1.01	(0.88, 1.15)		
Mainly meat	646	56,381	1.22	(1.13, 1.32)	485	45,269	1.26	(1.14, 1.38)	161	11,112	1.13	(0.96, 1.32)	0.0056	562	39,882	1.10	(1.01, 1.20)	359	28,425	1.08	(0.96, 1.20)	203	11,457	1.14	(0.99, 1.33)	0.2524	0.2598
**Smoking**																											
Never	4,048	304,176	1.00	Ref.	2,987	239,254	1.00	Ref.	1,061	64,922	1.00	Ref.		2,409	146,275	1.00	Ref.	1,586	104,348	1.00	Ref.	823	41,927	1.00	Ref.		
Former	1,525	108,631	1.08	(1.01, 1.14)	1,063	83,062	1.05	(0.98, 1.13)	462	25,569	1.14	(1.02, 1.27)		1,047	60,625	1.06	(0.98, 1.14)	695	41,982	1.10	(1.01, 1.21)	352	18,643	0.98	(0.86, 1.12)		
Current	5,132	443,431	1.05	(1.001, 1.093)	3,691	346,909	1.03	(0.98, 1.09)	1,441	96,522	1.08	(0.99, 1.18)	0.1138	2,918	198,331	1.08	(1.02, 1.15)	1,927	141,953	1.10	(1.03, 1.18)	991	56,378	1.05	(0.95, 1.16)	0.1719	0.0065
**Alcohol consumption**																											
None	3,832	256,821	1.00	Ref.	2,859	204,661	1.00	Ref.	973	52,160	1.00	Ref.		1,949	112,249	1.00	Ref.	1,365	81,944	1.00	Ref.	584	30,305	1.00	Ref.		
≤twice/week	4,842	477,804	0.70	(0.83, 0.91)	3,605	379,168	0.88	(0.84, 0.93)	1,237	98,636	0.84	(0.77, 0.92)		3,199	231,771	0.93	(0.87, 0.99)	2,151	166,921	0.91	(0.85, 0.98)	1,048	64,850	0.97	(0.87, 1.08)		
≥3 times/week	2,031	121,602	0.97	(0.92, 1.03)	1,277	85,396	0.95	(0.89, 1.02)	754	36,206	1.00	(0.90, 1.11)	0.1175	1,226	61,212	1.07	(0.996, 1.157)	692	39,419	0.99	(0.90, 1.09)	534	21,793	1.24	(1.09, 1.40)	0.0074	0.0012
**Physical activity**																											
None	5,640	420,436	1.00	Ref.	4,038	327,068	1.00	Ref.	1,602	93,368	1.00	Ref.		2,885	178,379	1.00	Ref.	1,875	126,173	1.00	Ref.	1,010	52,206	1.00	Ref.		
≤twice/week	3,049	288,425	0.94	(0.90, 0.98)	2,210	227,127	0.94	(0.89, 0.99)	839	61,298	0.95	(0.87, 1.03)		2,147	146,013	0.99	(0.93, 1.05)	1,431	104,712	1.00	(0.93, 1.07)	716	41,301	0.98	(0.88, 1.08)		
≥3 times/week	2,016	147,366	1.00	(0.96, 1.05)	1,493	115,030	1.02	(0.96, 1.09)	523	32,336	0.93	(0.84, 1.02)	0.0519	1,342	80,840	0.97	(0.91, 1.03)	902	57,399	1.00	(0.92, 1.08)	440	23,441	0.91	(0.82, 1.02)	0.1819	0.0062
**Women**																											
**Meal type**																											
Balanced	5,934	538,212	1.00	Ref.	4,789	462,550	1.00	Ref.	1,145	75,662	1.00	Ref.		2,704	128,447	1.00	Ref.	1,993	98,112	1.00	Ref.	711	30,335	1.00	Ref.		
Mainly vegetables	2,521	180,333	1.04	(0.99, 1.09)	2,037	152,950	1.05	(0.996, 1.110)	484	27,383	1.01	(0.91, 1.13)		857	37,548	0.99	(0.91, 1.07)	615	28,662	0.96	(0.88, 1.05)	242	8,886	1.06	(0.92, 1.23)		
Mainly meat	270	36,100	1.13	(0.999, 1.280)	222	31,920	1.10	(0.96, 1.26)	48	4,180	1.28	(0.95, 1.71)	0.2524	98	6,330	1.00	(0.82, 1.23)	59	4,947	0.84	(0.65, 1.10)	39	1,383	1.38	(0.997, 1.909)	0.0125	0.0833
**Smoking**																											
Never	8,262	717,103	1.00	Ref.	6,680	615,075	1.00	Ref.	1,582	102,028	1.00	Ref.			3,506	164,815	1.00	Ref.	2,552	125,756	1.00	Ref.	954	39,059	1.00	Ref.		
Former	126	13,160	1.16	(0.97, 1.39)	108	11,681	1.19	(0.98, 1.44)	18	1,479	1.01	(0.64, 1.62)			50	2,464	1.26	(0.95, 1.68)	40	1,999	1.36	(0.99, 1.87)	10	466	1.00	(0.54, 1.88)		
Current	337	24,383	1.24	(1.11, 1.39)	260	20,664	1.24	(1.09, 1.40)	77	3,719	1.25	(0.99, 1.58)	0.5023		103	5,046	1.08	(0.89, 1.32)	75	3,967	1.08	(0.85, 1.36)	28	1,079	1.10	(0.75, 1.62)	0.3507	0.2176
**Alcohol consumption**																												
None	6,805	508,073	1.00	Ref.	5,472	432,402	1.00	Ref.	1,333	75,671	1.00	Ref.		2,911	128,373	1.00	Ref.	2,121	97,844	1.00	Ref.	790	30,529	1.00	Ref.		
≤twice/week	1,727	231,517	0.88	(0.84, 0.93)	1,429	202,701	0.88	(0.83, 0.94)	298	28,816	0.89	(0.78, 1.01)		658	39,971	0.99	(0.91, 1.08)	486	30,941	1.00	(0.91, 1.11)	172	9,030	0.97	(0.81, 1.15)		
≥3 times/week	193	15,055	1.03	(0.89, 1.19)	147	12,317	1.04	(0.88, 1.23)	46	2,738	1.00	(0.74, 1.35)	0.8231	90	3,982	1.08	(0.88, 1.34)	60	2,937	1.05	(0.81, 1.36)	30	1,045	1.17	(0.81, 1.69)	0.4683	0.0038
**Physical activity**																											
None	5,623	496,086	1.00	Ref.	4,516	425,432	1.00	Ref.	1,107	70,654	1.00	Ref.		2,398	108,134	1.00	Ref.	1,714	82,156	1.00	Ref.	684	25,978	1.00	Ref.		
≤twice/week	1,600	149,271	0.99	(0.93, 1.05)	1,319	128,770	1.00	(0.94, 1.06)	281	20,501	0.95	(0.83, 1.08)		636	34,559	0.92	(0.84, 1.01)	480	26,543	0.96	(0.87, 1.06)	156	8,016	0.83	(0.69, 0.99)		
≥3 times/week	1,502	109,287	1.02	(0.96, 1.08)	1,213	93,217	1.01	(0.95, 1.08)	289	16,070	1.06	(0.93, 1.21)	0.2617	625	29,633	0.91	(0.836, 0.997)	473	23,022	0.93	(0.84, 1.03)	152	6,611	0.87	(0.73, 1.03)	0.1090	0.0714

^a^HRs were adjusted for age, sex (for total subjects only), and income level, and additionally mutually adjusted for lifestyle-related risk factors (all categorical variables) except BMI.

^b^HRs were adjusted for age, sex (for total subjects only), and income level, and additionally mutually adjusted for lifestyle-related risk factors (all categorical variables) except BMI and FBG.

^c^P_interaction_: p value of interaction between lifestyle-related factor and FBG within BMI level

^d^P_interaction_: p value of interaction between lifestyle-related factor and BMI level

Abbreviations: HR, hazard ratio; CI, confidence interval; BMI, body mass index; FBG, fasting blood glucose

The association between lifestyle-related factors and IHD risk was modified by BMI. Significant interactions were observed between BMI and alcohol consumption (p = 0.0002) and between BMI and physical activity (p = 0.0063). The interaction of physical activity with BMI was not significant in women. The interaction between smoking status and BMI was significant in men (p = 0.0065). Regarding the interaction between FBG level and lifestyle-related factors, the association between meal type and IHD risk was statistically different by FBG level in both BMI<25 kg/m^2^ (p = 0.0106) and BMI≥25 kg/m^2^ (p = 0.0281), particularly in men with BMI<25 kg/m^2^ (p = 0.0056) and women BMI≥25 kg/m^2^ (p = 0.0125). A statistically significant interaction between alcohol consumption and FBG level was observed in total subjects (p = 0.0118) and in men (p = 0.0074) with BMI ≥25 kg/m^2^.

## Discussion

This study showed that lifestyle-related factors were significantly associated with IHD incidence in both men and women, and the effects varied according to BMI and FBG levels among Korean adults. Subjects with a high BMI, high FBG level, mainly meat consuming meal type, or former or current smoking showed an elevated IHD incidence, whereas those with alcohol consumption ≤twice/week or physical activity ≤twice/week had a reduced IHD risk for both sexes. As the numbers of lifestyle-related risk factors increased, the risk of IHD was also significantly increased. This increased risk was more pronounced in women than in men, with a significant sex interaction. Significant interactions between BMI and alcohol consumption (p = 0.0002) and between BMI and physical activity (p = 0.0063) were observed for IHD incidence in all subjects. Interactions between FBG level and meal type and between FBG level and alcohol consumption were observed.

A high BMI was the strongest risk factor of IHD incidence in our study. In a previous Korean study on BMI and IHD incidence, young adults with obesity grade 1 (BMI 25.0–29.9 kg/m^2^) showed a significant increase in IHD risk (45% in men and 52% in women) compared with the reference group (BMI 18.5–22.9 kg/m^2^) [40].

This study reported higher IHD incidence rates than our results (29% in men and 30% in women), potentially because they excluded only IHD patients at baseline [40], while our study excluded IHD patients and other patients with circulatory diseases or T2D who were more likely to develop IHD.

A high FBG level was also a significant risk factor of IHD in our study, which is consistent with the results of previous studies [12, 13]. T2D (FBG≥126 mg/dL) has been confirmed to increase IHD incidence [41–43]. A meta-analysis of 17 prospective studies worldwide showed a positive relationship between IHD risk and impaired fasting glucose (FBG 100–125 or 110–125 mg/dL) [13].

Previous studies in Western countries reported that high meat intake increased IHD risk in both men and women [17, 21, 44, 45]. However, in our study, women consuming mainly meat did not show a significant increase in IHD risk. The traditional Korean diet is rich in vegetables, while Western dietary patterns comprise food with more fat content [46]. Moreover, women tend to eat more fruits, vegetables, and fiber than men [47]. Considering the healthier diet of women and the Korean diet of various vegetables, we assume that the three meal types used in our study are not sufficient to fully distinguish a mainly meat diet from a balanced diet, which explains why a mainly meat diet among women did not induce a significant change in IHD risk.

In this study, women current smokers showed increased IHD risk by 20% compared with women who were never smokers, while the risk was increased by 6% among men current smokers. A previous meta-analysis reported that smoking increased IHD risk more adversely in women than in men [48]. Smoking may have more harmful effects on IHD in women because women smokers tend to have a unhealthier cholesterol status, that is, a higher low-density lipoprotein cholesterol (LDL-C) level and a lower HDL-C level, than men smokers, and thus unhealthy cholesterol status could make blood vessel narrower and aggravate IHD risk [49, 50]. Information on LDL-C and HDL-C levels was not available in our study based on the 2003–2006 health examination. Thus, further research needs to investigate associations between sex disparity in Korean smokers and IHD incidence with more various metabolic indices including LDL-C and HDL-C levels.

Our results on alcohol consumption are consistent with those of previous studies advocating that only moderate alcohol consumption is inversely related to IHD risk [16, 18]. Although some studies suggested that heavy alcohol consumption ≥5 times/week also reduced IHD incidence [17, 22], we did not observe such relationship in our study. Koreans tend to consume meat with alcohol [46, 51]; thus, the effect of alcohol consumption on IHD risk must be considered when studying dietary patterns among Koreans. High-fat-content food like meat could exacerbate IHD risk especially with heavy drinking.

Moderate physical activity has been shown to decrease IHD risk in both men and women in previous studies [21, 44, 52]. However, in our study, women with any physical activity did not show a significant decrease in IHD risk. According to a meta-analysis on the dose-response relationship between physical activity and IHD risk, physical activity in women was inversely related to IHD risk, and the advantageous effect was greater in women than in men [52]. Our results, however, showed the opposite, in that only men with physical activity ≤twice/week had a significantly decreased HR of IHD. More research is necessary on why women in our study did not show any significant relationship between physical activity and IHD risk.

In this study, subjects with any number of lifestyle-related risk factors showed a significantly increased IHD risk in both sexes. Noticeably, the difference in IHD risk with 3 or more lifestyle-related risk factors compared to no risk factors was significantly higher in women than in men. However, the opposite was seen in a Japanese study reporting that only Japanese men with a high FBG or a high BMI showed a significantly increased IHD risk, but not in women [53]. This disparity in the results of the two studies might be due to the different selection of subjects by age, and by risk factors, except high BMI and high FBG levels. Our subjects were aged over 20 years, while subjects in the Japanese study were aged between 40–69 years. To our knowledge, the result of our study has not been fully explained by previous research, and there has been no study on IHD risk considering lifestyle-related factors with BMI and FBG levels. In this regard, future studies need to clarify why women showed more adverse effects from increasing numbers of lifestyle-related risk factors.

This study has some limitations. First, the NSC-v2 of the NHIS might contain some information bias due to a possible gap between recorded diseases and actual diseases of the subjects, since the disease codes were entered for the purpose of health insurance reimbursement. Some diseases might have been overestimated considering the intention of receiving larger reimbursement. This might have affected our inclusion/exclusion of subjects. Second, there was uncertainty in selecting patients with preexisting diseases due to unavailability of information before 2002, although we considered health examination data in 2002 as part of the washout period. Third, a self-report of lifestyle-related factors might lead to response bias. We were not able to use information on amount of alcohol consumption and intensity of physical activity since these were not collected at baseline. Additionally, we did not consider changes in lifestyle-related factors during the follow-up periods. Lastly, there could be additional confounding factors such as waist circumference and blood cholesterol levels that were not available in the NSC-v2.

Despite these limitations, this study has the advantages. To our knowledge, there is no research regarding the effect of multiple lifestyle-related factors on IHD incidence between sexes according to the status of BMI and FBG. To address this research gap, our study estimated the difference in IHD risk between men and women according to lifestyle-related factors stratified by BMI and FBG levels. Furthermore, we analyzed the dose-response relationship between IHD risk and lifestyle-related risk factors and estimated PARs. Therefore, our results could provide information for developing specific lifestyle guidelines to prevent IHD among Korean men and women according to BMI and FBG levels. Our results can serve as reference for other Asian countries with similar lifestyle patterns and for Western countries with different obesity standards.

## Conclusions

The current study shows that the risk of IHD is higher in people with more lifestyle-related risk factors such as mainly vegetable/mainly meat diet, former/current smoking, alcohol consumption ≥3 times/week, and no physical activity and these factors are influenced by BMI and FBG level among Korean adults. The number of lifestyle-related factors and their effect on IHD incidence was more pronounced in women than in men. This suggests that programs to prevent IHD should consider lifestyle and sex along with BMI and FBG levels. Further research is necessary to investigate the effects of other metabolic indices and lifestyle-related risk factors on the risk of IHD.
